# Hedonic and autonomic responses in promoting affective touch

**DOI:** 10.1038/s41598-023-37471-9

**Published:** 2023-07-11

**Authors:** Alessandro Mazza, Monia Cariola, Francesca Capiotto, Matteo Diano, Selene Schintu, Lorenzo Pia, Olga Dal Monte

**Affiliations:** 1 Department of Psychology, University of Turin, Via Verdi 10, Turin, Italy; 2 Center for Mind/Brain Sciences-CIMeC, University of Trento, Rovereto, Italy; 3 Department of Psychology, The George Washington University, Washington, DC USA; 4 Department of Psychology, Yale University, New Haven, CT USA

**Keywords:** Neuroscience, Social behaviour, Social neuroscience

## Abstract

Interpersonal touch is intrinsically reciprocal since it entails a person promoting and another receiving the touch. While several studies have investigated the beneficial effects of receiving affective touch, the affective experience of caressing another individual remains largely unknown. Here, we investigated the hedonic and autonomic responses (skin conductance and heart rate) in the person promoting affective touch. We also examined whether interpersonal relationship, gender, and eye contact modulate these responses. As expected, caressing the partner was perceived as more pleasant than caressing a stranger, especially if the affective touch occurred together with mutual eye contact. Promoting affective touch to the partner also resulted in a decrease of both autonomic responses and anxiety levels, suggesting the occurrence of a calming effect. Additionally, these effects were more pronounced in females compared to males, indicating that hedonic and autonomic aspects of affective touch are modulated by both social relationship and gender. These findings show for the first time that caressing a beloved one is not only pleasant but also reduces autonomic responses and anxiety in the person promoting the touch. This might suggest that affective touch has an instrumental role for romantic partners in promoting and reinforcing their affective bonding.

## Introduction

Touch is the simplest and most direct of all sensory systems^1^. It has been described as the paramount means of interpersonal exchange, which plays a crucial role in emotional processing, social interactions, and cognition^2^. The skin has been defined as “a social organ”^3^ and whether touch comes from a firm handshake, an encouraging pat on the shoulder, or a gentle caress, it has a strong and direct nonverbal communicative function^4^. The pleasant effects of social touch are mainly determined by a particular type of touch called *affective touch*; a touch resembling a caress, essential for emotions’ communication and social bonds^4^. The *affective touch* is a standalone type of tactile experience that relies on the hyper-specialized somatosensory system, called C-Tactile (CT) afferent system, which is activated by slow and gentle strokes^5,6^ and a temperature close to the one of the human skin^7^. However, differently from the unequivocal nature of discriminative touch, *affective touch* can have a positive or a negative valence depending on, for example,  the speed and body’s area in which the touch occurs^8,9^, socio-cultural norms, context, gender^10^, interpersonal relationship, identity of the person providing the touch^3,11,12^,and nonverbal visual cues^13^.

Research conducted across the last few decades has come to the agreement that the pleasant effects produced by *affective touch* vary as a function of the relationship between individuals. Undoubtedly, more frequent physical contact and closeness are observed among romantic partners than strangers. Being touched by one's partner lowers arousal levels by reducing the activity of brain areas involved in alarm processing^14,15^, promotes recovery following a stressful event^16^, diminishes pain perception^17^, and prompts physiological coupling between partners^18^. On the contrary, being touched by a stranger does not produce the calming and analgesic effects observed between partners and it can rather induce states of anxiety and discomfort^12,19^. Indeed, an unexpected touch from a stranger is likely to be experienced as discomforting and unpleasant^20^.

Another relevant aspect reported to modulate the hedonic experience of *affective touch* is the gender of the subjects involved in the social interaction. Russo et al.^21^ have reported a gender asymmetry in the evaluation of *affective touch* with females showing higher sensitivity and accuracy in communicating feelings and emotions via touch than men. For example, females tend to find less pleasant a touch from a male stranger than a male friend, while men are equally comfortable with a touch from either a woman stranger or a woman friend^22^. Nonetheless, studies on the modulatory effect of gender on *affective touch* are dated^23^, often inconsistent^24^ and mainly based on participants’ subjective rating.

Recent studies have also pointed out the possible contribution of visual cues as a factor that might come into play during *affective touch*^12,25^. Among several social cues, eye contact has been considered a rich source of social information that promotes social interactions in both human^26^ and non-human primates^27^. Eye contact has been argued to play a leading role in strengthening emotional sharing between individuals and evoking positive affective reactions^28^. Nevertheless, the meaning of eye contact is subordinated to both contextual factors and interpersonal relationships^29,30^; an eye contact with a familiar person, but not with a stranger, can enhance affection, attention, and social inclusion^31^. Although eye contact is a key feature of social interaction and in daily life *affective touch* often occurs together with eye contact exchange, only a few studies have assessed the link between eye gaze and touch^32,33^, thus  their relationship is still  largely unexplored.

Some studies have also investigated the hedonic and sensorial aspects of *affective touch* from the perspective of the person promoting it (hereafter also referred to as the giver). For example, another person’s skin is perceived as more pleasant to touch^34^ as well as softer^35,36^ than one’s own, a phenomenon called “social softness illusion”. Crucially, this effect only occurs when tactile contact resembles *affective touch*^36^, which in turn is perceived as pleasant and rewarding by the stroked person^8^. These studies highlight how the pleasantness of stroking others’ skin might play a central role in promoting and strengthening interpersonal bonding. However, the pleasantness of promoting *affective touch* might change depending on contextual social cues such as the interpersonal relationship with the receiver, the giver’s gender, and the presence of a visual feedback between the two interacting individuals. In the last decade, several researchers have shown the influence of interpersonal relationship, gender, and eye contact on the person receiving *affective touch*, but  how these factors drive the hedonic and autonomic responses on the person promoting *affective touch* has been largely neglected.

To fill in this gap, with a series of experiments, we investigated the hedonic and physiological responses on the person promoting a slow (1–10 cm/s)^37^ and gentle *affective touch* (CT-optimal touch) over the receiver’s forearm. In Experiment 1, we examined whether the interpersonal relationship (Partner vs Stranger), gender (Male vs Female), and visual feedback (Eye Contact vs Non-eye Contact) could modulate both the subjective experience and the autonomic responses of the giver. We measured hedonic responses while concomitantly recording electrodermal and cardiac activity as indices of autonomic arousal associated to affective states^38,39^. Similarly to what has been already observed when people receive *affective touch*^40,41^, we expected caressing the partner to be perceived as more pleasant than caressing a stranger. Also, an eye contact with a close person is known to carry positive valence^31^, thus we hypothesized that the pleasantness of caressing the partner would be enhanced when the touch occurred during an eye contact exchange. In line with these hypotheses, we also predicted that stroking a stranger would result in enhanced stress and anxiety and would be associated with higher autonomic arousal. Additionally, we also predicted that both the receiver’s identity and the presence of eye contact would modulate physiological arousal. We expected to observe larger skin conductance responses with a stranger than with the partner especially if the *affective touch* occurred together with mutual eye contact.  Finally, given several pieces of evidence showing that females are more sensitive than males to a stranger’s touch^21,42^, we hypothesized that these effects could vary as a function of the giver’s gender, with females experiencing more anxiety and larger autonomic responses than males. Given that skin conductance is known to respond to eye contact ^43,44^ and to display anticipatory activity^45^, to exclude that the physiological responses observed in Experiment 1 could have been driven by eye contact alone or by any anticipatory effects, we conducted Experiment 2 in which we manipulated these two variables. In Experiment 2 we first assessed whether *affective touch* combined with eye contact produced a larger autonomic response than eye contact alone, then if such effect was larger with a stranger compared to the partner, and finally whether there were any gender differences. Moreover, to control  that the increase in skin conductance observed during *affective touch* was related to touch and not driven by any anticipatory effects due to the instructions participants received, we varied the instructions’ timing and removed any count-down preceding the touch so that participants could not predict its beginning. We hypothesized a stronger autonomic response when participants were engaged in *affective touch* as compared to just mutual eye contact, as well as a higher hedonic experience when interacting with their partners as compared to a stranger. Additionally, we predicted that the increase in skin conductance during *affective touch* would have been both independent from and larger than the physiological activity during the instructions period, thus ruling out the possibility that the increase in skin conductance observed during *affective touch* could be driven by an anticipatory effect.

## Results

### Experiment 1

**Figure 1:**
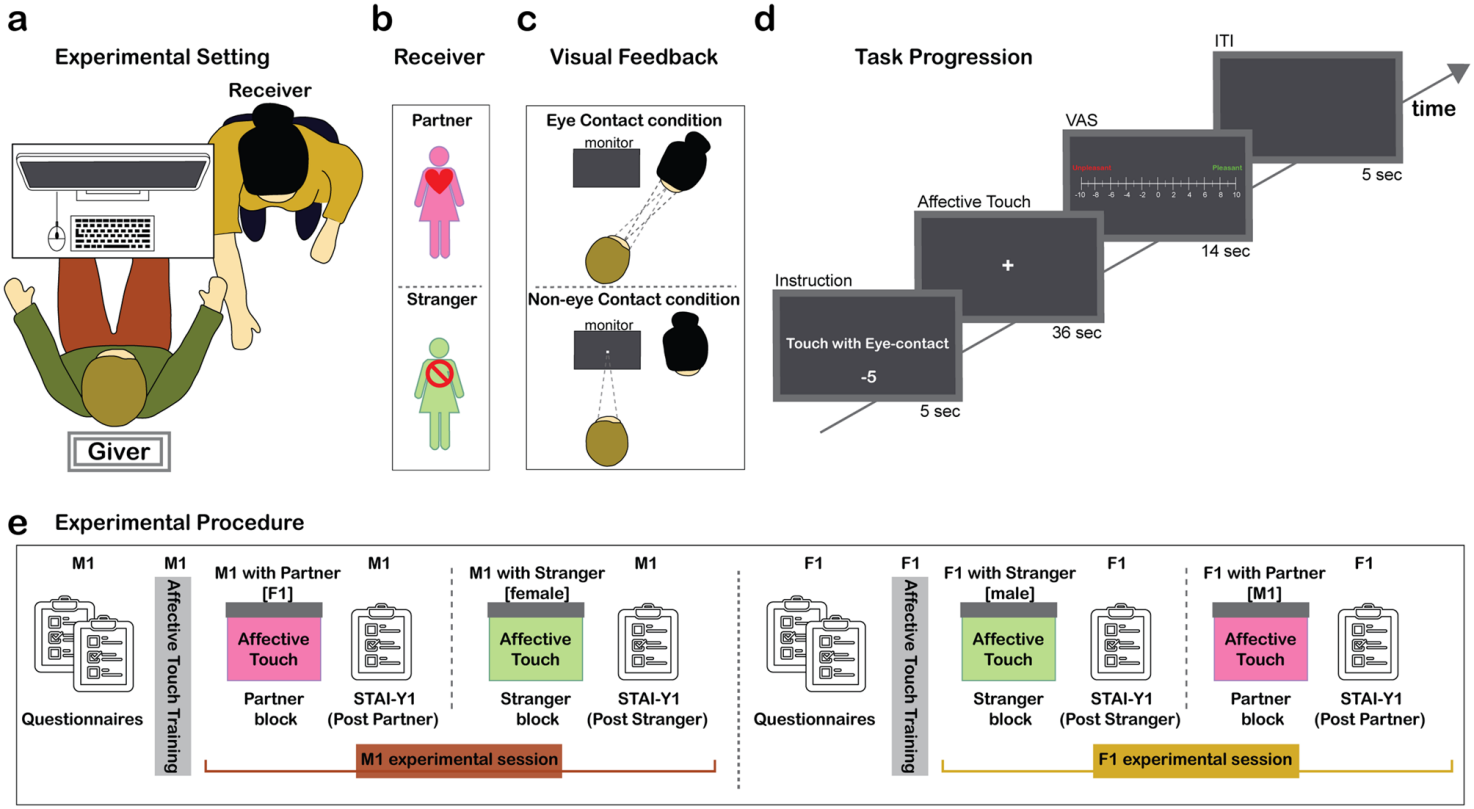
Experimental setting, variables, and task progression. (**a**) Experimental setting: the participants sat facing each other. Only the giver had a computer screen in front of him/her, showing trial-by-trial instructions. The giver gently stroked the right arm of the receiver (partner or stranger) with his/her dominant hand. (**b**) Receiver’s identity: the experiment consisted of two blocks, one with the giver’s partner (top) and one with a stranger played by a confederate (bottom). (**c**) Visual feedback: at the beginning of each trial in each block, participants were asked to promote *affective touch* while either exchanging a mutual eye contact with the receiver (Eye Contact condition; top) or looking at a fixation cross on the screen (Non-eye Contact condition; bottom). The two conditions were presented in a pseudo-randomized order. (**d**) Task progression: each block consisted of 18 trials lasting 1 min each: 5 s of instructions (i.e., promote the *affective touch* with or without eye contact); 36 s of *affective touch*; 14 s of pleasantness rating on a scale from −10 (unpleasant) to +10 (pleasant); and 5 s of inter-trial interval (ITI; return to baseline). (**e**) Experimental procedure: illustrated an example sequence of one experimental session for both a Male subject (M1) and a Female subject (F1). At the beginning of each experimental session, subjects were invited to fill in four questionnaires including the STAI-Y1 (see “Methods” section). Before the beginning of the experimental session, M1 (i.e., the male giver) underwent a brief *affective touch* training, then he completed the partner block with his female partner. At the end of this block, M1 was invited to fill in the STAI-Y1 questionnaire again (*Post* *Partner*). After a break, M1 performed a second block with a stranger (female confederate) and was invited to fill in the STAI-Y1 a third and last time (*Post* *Stranger*). After a longer break, F1 (M1’s partner) performed the same procedure as M1. She started with *affective touch* training, then she completed her first block with a stranger (male confederate) and after that she was invited to fill in the STAI-Y1 (*Post Stranger*). After a short break, F1 performed a second block with her partner (M1) then was invited to fill in the STAI-Y1 a third and last time (*Post Partner*). The subject starting (M1 or F1) and the order of the experimental blocks were counterbalanced across subjects.

Fifty participants (25 females) engaged in an ecological interactive *affective touch* paradigm (Fig. 1a) while electrodermal and electrocardiac activities were recorded from the person promoting *affective touch *(i.e., the giver). The experimental session included two blocks. In one block, the giver was invited to promote a slow and gentle *affective touch* (approximately 4 cm/s) over his/her partner’s forearm; in the other block, the giver was invited to promote the same type of *affective touch* over a stranger’s forearm (an opposite-gender confederate, i.e., an experimenter) (Fig. 1b). Since we were also interested in investigating the impact of mutual eye contact on *affective touch,* we employed two conditions: Eye Contact condition and Non-eye Contact condition (Fig. 1c), randomized within each block. As represented in Fig. 1d, at the beginning of each trial the giver was presented on the computer screen with the instructions for the upcoming touch together with a 5 s count-down. Then, the giver was invited to promote *affective touch *to the receiver that lasted 36 s (6 consecutive strokes). In the Eye Contact condition, the eye contact lasted for the whole trial duration (36 s). Next, a Visual Analog Scale (VAS) was presented on the screen and the subject was asked to rate the pleasantness of the touch he/she had promoted. At the end of each block participants were asked to fill in the STAI-Y1 questionnaire to assess changes in their anxiety levels after promoting a touch to the partner (*Post Partner*) and a stranger (*Post Stranger*) as compared to the beginning of the experimental session (Fig. 1e). Before the beginning of the experimental session, participants underwent a brief training session to make them familiar with promoting a gentle stroke at an optimal speed for *affective touch* (i.e., 4 cm/s)^37^. A clear description of the experimental procedure is depicted in Fig. 1e.

#### Hedonic responses

**Figure 2:**
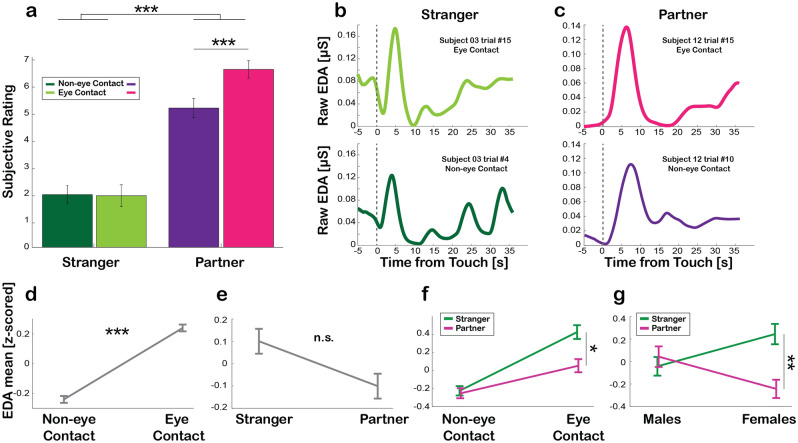
Hedonic and autonomic responses. (**a**) Bar plots show the mean pleasantness ratings reported by participants in the four conditions: *affective touch* given to a Stranger without Eye Contact (Non-eye Contact condition; dark green), to a Stranger with Eye Contact (Eye Contact condition; light green), to the Partner without Eye Contact (dark purple) and to the Partner with Eye Contact (light purple). Values shown are the mean ±  s.e.m. (**b**) Single-trace examples of raw electrodermal activity (EDA) aligned to the time of *affective touch* with a Stranger, with or without Eye Contact (light green on the top and dark green at the bottom, respectively). Vertical dotted grey line indicates the beginning of *affective touch* period (36 s duration). (**c**) Single-trace examples of raw EDA activity aligned to the time of *affective touch* with the Partner, with or without Eye Contact (light purple and dark purple, respectively), same format as (**b**). (**d**) Z-scored mean EDA values during *affective touch* delivered without Eye Contact (Non-eye Contact condition) and with Eye Contact (Eye Contact condition). (**e**) Z-scored mean EDA values during *affective touch* delivered to a Stranger and the Partner. (**f**) Z-scored mean EDA values when *affective touch* was delivered without Eye Contact (Non-eye Contact condition) and with Eye Contact (Eye Contact condition) separately for Stranger (green) and Partner (purple). (**g**) Z-scored mean EDA values when *affective touch* was delivered by Males or Females, separately for Stranger (green) and Partner (purple). Values shown are z-scored ± s.e.m. Significant results are indicated by asterisk * = p < 0.05; ** = p < 0.01; *** = p < 0.001. n.s. = not significant.

We first assessed whether the interpersonal relationship, gender, and eye contact could modulate the hedonic response on the person promoting *affective touch*. The mixed-factors analysis of the variance (ANOVA) on subjective rating (VAS scores on pleasantness) having Other (Partner vs Stranger) and Gaze (Eye Contact vs Non-eye Contact) as within-subject factors and Gender (Males vs Females) as between-subject factor, showed a main effect of Other [F_(1, 48)_ = 114.29, p < 0.001, η_p_^2^ = 0.705], indicating that participants rated as more pleasant to stroke their Partner than a Stranger. We also found a main effect of Gaze [F_(1, 48)_ = 21.49, p < 0.001, η_p_^2^ = 0.309], meaning that promoting *affective touch* to another person is rated as more pleasant when touch is accompanied by mutual Eye Contact compared to Non-eye Contact. Crucially, a significant interaction Other*Gaze [F_(1, 48)_ = 42.43, p < 0.001, η_p_^2^ = 0.469] indicated that mutual Eye Contact enhances the perceived pleasantness of *affective touch* compared to Non-eye Contact with the Partner [t_(49)_ = 7.60, p < 0.001] but not with a Stranger [t_(49)_ = 0.22, p = 0.83] (Fig. 2a). No other significant main effects or interactions were found.

These results, in line with our hypotheses, show that the hedonic experience of promoting *affective touch* is stronger when participants promote *affective touch* to their partners as compared to strangers and that such effect is enhanced when mutual eye contact occurrs during the interaction.

#### Autonomic (skin conductance) responses

We next investigated whether the differences observed at the hedonic level were reflected on the giver’s electrodermal activity (EDA) (Fig. 2b, c). The same mixed-factors ANOVA design used for VAS was run on EDA mean and showed a significant main effect of Gaze [F_(1, 48)_ = 107.02, p < 0.001, η_p_^2^ = 0.690; Fig. 2d], meaning a higher skin conductance level when *affective touch* is accompanied by mutual Eye Contact as compared to Non-eye Contact. Even though the main effect Other did not reach statistical significance [F_(1, 48)_ = 3.68, p = 0.061; Fig. 2e], we found a significant interaction Other*Gaze [F_(1, 48)_ = 12.48, p < 0.001, η_p_^2^ = 0.206; Fig. 2f] indicating that autonomic responses during *affective touch* vary as a function of both eye contact and the identity of the interacting person. In fact, only when *affective touch* was accompanied by Eye Contact, participants showed a higher level of skin conductance with a Stranger compared to their Partner [t_(49)_ = 2.62, p = 0.012]. This difference was instead absent during Non-eye Contact interactions [t_(49)_ = 0.28, p = 0.78]. Interestingly, we also observed a significant interaction Other*Gender [F_(1, 47)_ = 7.493, p = 0.009, η_p_^2^ = 0.135; Fig. 2g] indicating that only Females showed larger EDA mean when stroking a Stranger compared to the Partner [t_(24)_ = 2.69, p = 0.013]. This difference was not present in Male participants [t_(24)_ = 1.48, p = 0.15]. No other significant main effects or interactions were found.

These results show that  promoting *affective touch* to a stranger increases autonomic responses, and such effect is enhanced when mutual eye contact occurs during the interaction. Furthermore, as expected, the gender of the giver plays a significant role; females, but not males, show a greater increase in skin conductance when caressing a stranger compared to their partners.

#### Autonomic (heart rate) responses and subjective measures (self-report)

**Figure 3:**
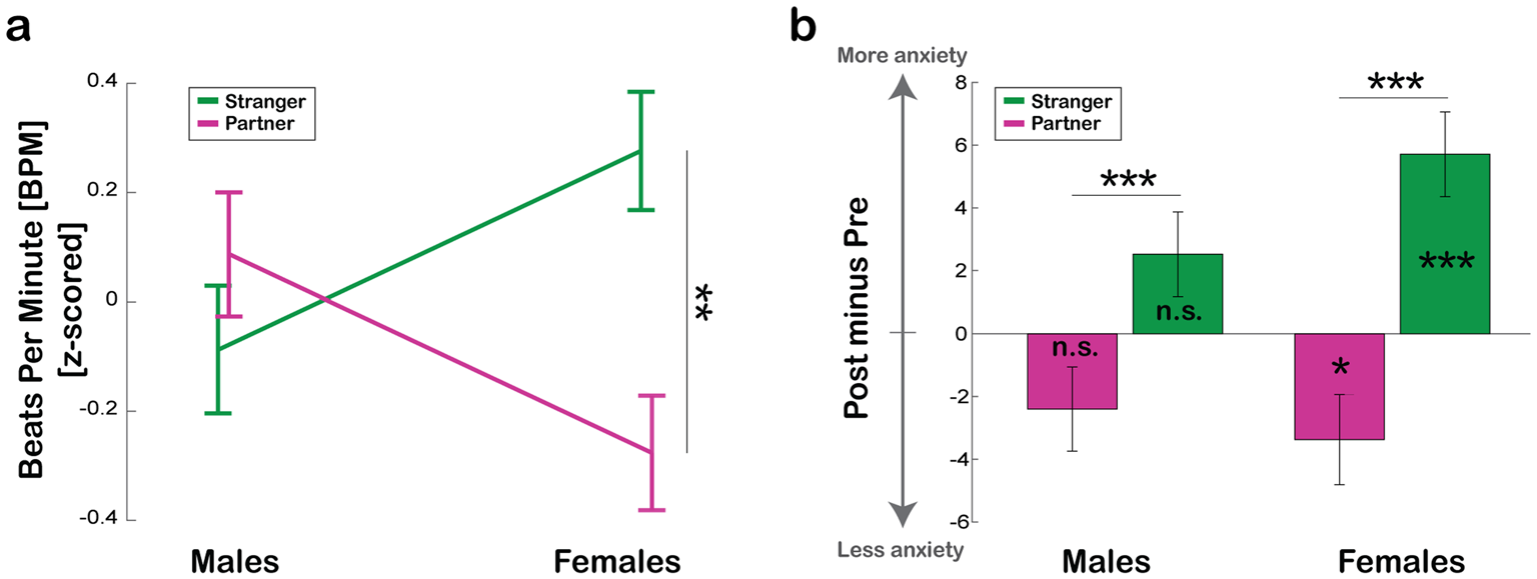
Heart rate and Self-reported anxiety levels. (**a**) Z-scored heart rate (Beats Per Minute; BPM) when *affective touch* was delivered by Males or Females to either a Stranger (green) or the Partner (purple)***.*** Heart rate was extracted considering the whole block duration, for both Partner and Stranger blocks. (**b**) State anxiety variations as measured by State-Trait Anxiety Inventory reported by Males and Females after completing the Stranger (green) and the Partner (purple) blocks; significance values depicted inside the bars indicate the p-values of t-tests against zero for each condition separately. Values shown are the mean ± s.e.m.. Significant results are indicated by asterisk * = p < 0.05; ** = p < 0.01; *** = p < 0.001. n.s. = not significant.

The mixed-factors ANOVA on heart rate (Beats Per Minute; BPM) averaged within each experimental block, with Other (Partner vs Stranger) as within-subject factor and Gender (Males vs Females) as between-subject factor, showed a significant Gender*Other interaction [F_(1, 46)_ = 8.79, p = 0.005, η_p_^2^ = 0.160; Fig. 3a]. This interaction, in line with our hypotheses and with skin conductance’s results, showed that only Female participants displayed a faster heart rate while promoting *affective touch* to a Stranger than to the Partner [t_(23)_ = 3.04, p = 0.006], while Male participants did not show any differences in heart rate depending on the receiver’s identity (t_(23)_ = 1.16, p = 0.26).

Next, using a self-report measure (State-Trait Anxiety Inventory-STAI-Y1) we assessed changes in anxiety levels related to *affective touch.* The mixed-factors ANOVA on the State-Trait Anxiety Inventory scores showed a main effect of Other [F_(1, 47)_ = 46.83, p =  < 0.001, η_p_^2^ = 0.499; Fig. 3b]. As expected, we found a general increase in anxiety levels after promoting *affective touch* to a Stranger (4.08 ± 0.97) [t_(48)_ = 4.19, p < 0.001] and a decrease after promoting it to the Partner (-2.88 ± 0.97) [t_(48)_ = − 2.6, p = 0.005]. These results confirm that promoting *affective touch* to the Partner produces calming effects, while promoting it to a Stranger enhances anxiety. Additionally, we found a significant interaction Other* Gender [F_(1, 47)_ = 4.14, p = 0.048, η_p_^2^ = 0.081]. Both Males [t_(24)_ = 3.98, p < 0.001] and Females [t_(23)_ = 5.53, p < 0.001] showed a significant difference between caressing a Stranger and the Partner; however, for Male participants neither promoting *affective touch* to the Partner nor to the Stranger significantly increased or decreased anxiety levels compared to their baseline [Δ_Partner_ = − 2.4 ± 1.34, t_(24)_ = − 1.79, p = 0.09; Δ_Stranger_ = 2.52 ± 1.35, t_(24)_ = 1.86, p = 0.08]. On the contrary, both effects were present in Females, for whom stroking the Partner produced significantly calming effects, while promoting *affective touch* to a Stranger raised their anxiety levels [Δ_Partner_ = − 3.38 ± 1.44, t_(23)_ = − 2.35, p = 0.028; Δ_Stranger_ = 5.71 ± 1.35, t_(23)_ = 4.23, p < 0.001]. These results confirm previous studies and our hypotheses. Given these results, we next asked whether differences observed at the hedonic and physiological levels between male and female subjects might have been driven by other traits. First of all, Males and Females did not differ in their anxiety level at baseline [Males 34.88 ± 1.13 and Females 33.96 ± 1.62; t_(47)_ = 0.401, p = 0.691]. Additionally, Males and Females did not differ in their personal attitude toward social situations involving touch in everyday life as measured by the STQ [Males 35.12 ± 2.29 and Females 34.17 ± 2.65; t_(48)_ = − 1.32, p = 0.193], nor in their level of distress when interacting with others as measured by the SIAS [Males 30.04 ± 2.50 and Females 29.04 ± 2.61; t_(48)_ = − 0.89, p = 0.373]. Moreover, neither the quality nor the strength of the relationship were perceived differently by Males and Females as measured by the DAS [Males 103.88 ± 1.50 and Females 102.96 ± 2.23; t_(48)_ = 0.31, p = 0.761] (see “Methods” section for details on questionnaires).

Overall, both skin conductance and heart rate measures reveal a larger autonomic activation in females compared to males when they had to promote *affective touch* to a stranger. Crucially, these findings reflect the STAI-Y1 questionnaire results, in which only females reported a significant increase in their level of anxiety with a stranger and a reduction of anxiety after promoting *affective touch* to their partner (see Supplementary Fig. 1 for an overview of the results).

### Experiment 2

**Figure 4:**
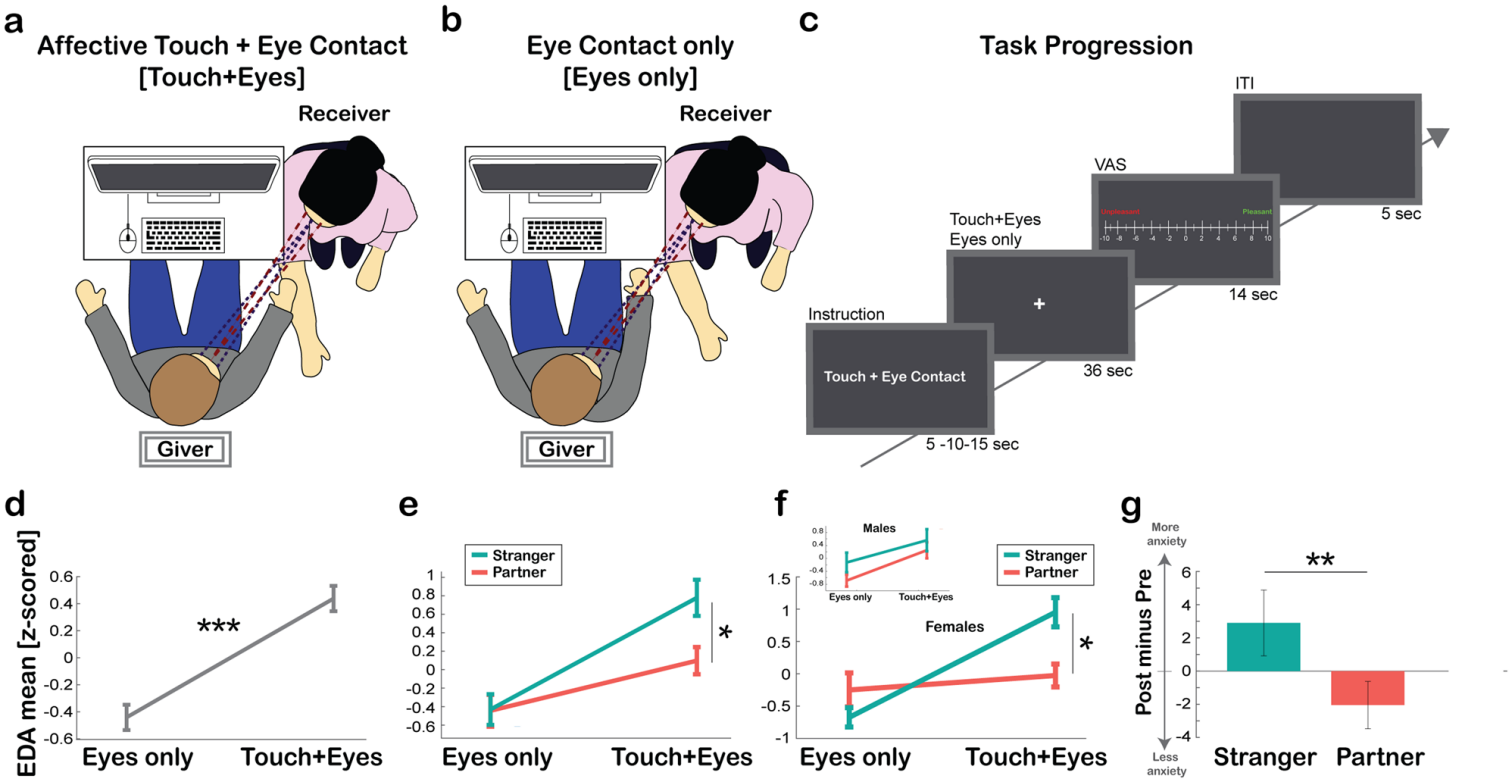
Experimental setting task progression and physiological responses. (**a**) Experimental setting: participants sat facing each other. Only the giver had a computer screen in front of him/her, showing trial-by-trial instructions. The giver promoted *affective touch* on the right arm of the receiver participant with his/her dominant hand while looking at him/her in the eyes (Touch + Eyes). The receiver was either his/her partner or a stranger played by a confederate. (**b**) Same format as in (**a**) but depicting the Eye Contact only condition (Eyes Only). (**c**) Task progression: each block consisted of 20 trials. The instructions presented on the screen had three different presentation times (5, 10 or 15 s), then at the end of the instructions a white cross appeared on the screen indicating the beginning of the experimental condition (i.e., Affective Touch + Eye Contact or Eye Contact only) and lasted for 36 s. After that, a VAS for pleasantness rating, ranging from −10 (unpleasant) to +10 (pleasant) was presented for 14 s, and followed by 5 s of inter-trial interval (ITI). (**d**) Z-scored mean EDA values during a mutual eye contact exchange (Eyes Only) and during and *affective touch* and eye contact (Touch + Eyes). (**e**) Z-scored mean EDA values during Eyes Only and Touch + Eyes separately for Stranger (light green) and Partner (light red). (**f**) Z-scored mean EDA values when Females (main plot) and Males (inset) engaged in a mutual eye contact exchange (Eyes Only) and in *affective touch* combined with an eye contact (Touch + Eyes) separately for the Stranger (light green) and the Partner (light red). (**g**) State anxiety variations after interacting with a Stranger (light green) and the Partner (light red). Values shown are the mean ± s.e.m. Significant results are indicated by asterisk * = p < 0.05; ** = p < 0.01; *** = p < 0.001.

To control that the hedonic and skin conductance responses observed in Experiment 1 were not driven by eye contact per se nor by any anticipatory effects linked to the trial’s instructions, in Experiment 2 (N = 18 participants; 10 Females), we manipulated the aforementioned two variables. We designed an experimental setting similar to Experiment 1, whereby in one block the giver promoted *affective touch* to the subject’s partner forearm while in the other block he/she stroked the forearm of a stranger (an opposite-gender confederate). To confirm that physiological responses in Experiment 1 were not only explained by the presence/absence of mutual contact exchange between the giver and receiver, but rather by *affective touch*, we employed two conditions: Affective touch + Eye Contact condition (Touch + Eyes, Fig. 4a) and Eye Contact only condition (Eyes only, Fig. 4b). These allowed us to keep eye contact fixed across conditions and manipulate the presence/absence of a concomitant *affective touch*. As we hypothesized that *affective touch* per se has an impact on the giver’s autonomic system, we expected to observe larger skin conductance responses when eye contact was coupled with *affective touch* than during eye contact alone. In both conditions, the eye contact lasted for the whole trial duration. Moreover, to confirm that the increase in skin conductance observed during *affective touch* was related to *affective touch* and not driven by any anticipatory effects, we varied the instructions timing (5, 10, or 15 s) and removed any countdown: participants were instructed to start the trial as soon as instructions disappeared from the screen, thus they were not able to predict the beginning of the touch (Fig. 4c).

#### Hedonic responses

The mixed-factors ANOVA on subjective rating having Other (Partner vs Stranger) and Touch (Touch + Eyes vs Eyes Only) as within-subject factors and Gender (Males vs Females) as between-subject factor showed a main effect of Other [F_(1, 16)_ = 53.16, p < 0.001, η_p_^2^ = 0.769], indicating that participants rated as more pleasant to stroke their Partner than a Stranger. We also found a significant interaction Other*Gender [F(1, 16) = 8.03, p = 0.012, ηp2 = 0.334] indicating that caressing their partner was perceived as more pleasant for Females than Males [t_(16)_ = − 1.95, p = 0.035]. Crucially, a significant interaction Other*Touch [F_(1, 16)_ = 15.97, p = 0.001, η_p_^2^ = 0.499] indicated that *affective touch* when combined with a mutual Eye Contact was perceived as more pleasant than just an Eye Contact only with the Partner [t_(17)_ = 5.95, p < 0.001] but not with a Stranger [t_(17)_ = 1.29, p = 0.215]. No other significant effects or interactions were found.

These results, in line with those reported in Experiment 1, show that participants perceived as more pleasant to interact with their partners as compared to strangers. Crucially, the results also suggest that the pleasantness of promoting *affective touch* is enhanced by eye contact, but only when interacting with the partner.

#### Autonomic (skin conductance) responses and subjective measures (self-report)

Next, we assessed whether *affective touch* produced a different skin conductance response with respect to eye contact only. We also aimed to investigate whether such effect was larger with a stranger compared to the partner and if there were any gender differences. Thus, we ran a mixed-factors ANOVA on EDA mean, with Other (Partner vs Stranger) and Touch (Touch + Eyes vs Eyes Only) as within-subject factors and Gender (Male vs Females) as between-subject factors. We found a significant main effect of Touch [F_(1, 16)_ = 20.27, p < 0.001, η_p_^2^ = 0.559], with Touch + Eyes condition showing larger values than the Eyes Only condition, suggesting that *affective touch* is accompanied by a larger skin conductance response than eye contact only (Fig. 4d). We also found a significant interaction Other*Touch [F_(1, 16)_ = 4.66, p = 0.046, η_p_^2^ = 0.225], indicating that the autonomic responses vary as a function of the receiver’s identity. Promoting *affective touch* to a Stranger compared to the Partner resulted in an increase of autonomic response [t_(17)_ = 2.33, p = 0.034] whereas this difference was absent during Eyes Only condition [t_(17)_ = 0.05, p = 0.964], indicating that larger EDA values with a Stranger than with the Partner were observed only when *affective touch* was involved, and not with eye contact alone (Fig. 4e). Lastly, we found a significant triple Other*Touch*Gender interaction [F_(1, 16)_ = 9.64, p = 0.007, η_p_^2^ = 0.376] (Fig. 4f). To disentangle this triple interaction, we ran two separate ANOVAs, one for Males and one for Females respectively, with Other (Partner vs Stranger) and Touch (Touch + Eyes vs Eyes Only) as within-subject factors. For Male participants we only found a significant main effect of Touch [F_(1, 7)_ = 5.60, p = 0.050, η_p_^2^ = 0.445], with Touch + Eyes condition showing larger EDA values than the Eyes Only condition (Fig. 4f inset). As for Male participants, for Female participants we found a significant main effect of Touch [F_(1, 9)_ = 19.30, p = 0.002, η_p_^2^ = 0.682], with Touch + Eyes condition showing larger values than Eyes Only condition. However, we also found a significant Other*Touch interaction [F_(1, 9)_ = 37.68, p < 0.001, η_p_^2^ = 0.807], showing that a significant difference in EDA response between Partner and Stranger was present only for the Touch + Eyes condition [t_(9)_ = − 2.82, p = 0.020], and not for the Eyes Only condition [t_(9)_ = 1.14, p = 0.285]. This interaction suggests that larger EDA values with the stranger are observed only when *affective touch* is present, and not with eye contact alone (Fig. 4f). Moreover, in line with the findings of Experiment 1, participants reported higher levels of state anxiety after interacting with a Stranger compared to their Partner [F_(1, 16)_ = 8.83, p = 0.009] (Fig. 4g). No other significant effects or interactions were found.

Overall, these results show that *affective touch* produces a larger skin conductance response than eye contact alone, thus confirming our hypothesis that the effects observed in Experiment 1 were not merely driven by mutual eye contact. Additionally, with Experiment 2 we replicated the results reported in Experiment 1. We found that a larger autonomic response occured when *affective touch* is given to a stranger compared to the partner and that this difference is enhanced with mutual eye contact, with stronger effects in female compared to male participants (see Supplementary Fig. 2 for an overview of the results).

Finally, we also demonstrated that skin conductance responses occurring during *affective touch* were both independent from and larger than during the instructions period, thus ruling out any anticipatory effect (see Supplementary Results and Supplementary Fig. 3).

## Discussion

*Affective touch* plays a key evolutionary role in socio-emotional interactions, produces calming effects, promotes social bonding, and strengthens affiliative behaviors^4,46,47^. In the present study, our primary goal was to examine the hedonic experience and the autonomic responses on the person promoting *affective touch.* Specifically, we were interested in investigating whether and to which extent the relationship between the giver and the receiver, the giver’s gender, and the occurrence of mutual eye contact could modulate hedonic and autonomic responses during *affective touch.* We found that participants reported as more pleasant caressing their partners than a stranger and that this effect was enhanced when mutual eye contact occurred during the interaction. At the physiological level we observed that the skin conductance responses varied not only based on the interpersonal relationship between the two interacting participants (partner vs stranger), but also as a function of both the giver’s gender and the exchange of eye contact. Indeed, the difference in skin conductance between partner and stranger, with higher skin conductance activity when stroking a stranger compared to the partner, was enhanced when mutual eye contact occurred during the interaction. Additionally, we found that females showed a greater increase in skin conductance when caressing a stranger compared to caressing their partners, while this difference was not present for male participants.

These results show for the first time that *affective touch* is accompanied by context-specific hedonic experiences and elicits autonomic reactions in the person promoting it, and not only in the person receiving it as the existing literature has previously shown. These findings also suggest that different social variables, such as giver’s identity, gender, and eye contact are encoded by the autonomic nervous system and modulate the physiological responses in the person promoting *affective touch*. Moreover, with Experiment 2 we showed that  the skin conductance increase during *affective touch* was both independent from and larger than the one measured during the instructions period, thus ruling out the possibility that the enhancement in skin conductance observed during *affective touch* was driven by an anticipatory effect.

### Subjective responses

At the hedonic level we found that promoting *affective touch* to the partner was perceived as more pleasant than promoting it to a stranger. This finding goes beyond previous studies which found that hetero-directed touch is more pleasant than self-directed touch^34,36^ and reveals that the pleasantness of a hetero-directed *affective touch* depends on the type of relationship between the giver and the receiver^15^. From the receiver’s point of view, it has been reported that the degree of the pleasantness of receiving *affective touch* can vary as a function of the type of relationship; for instance, people are less comfortable being touched by a friend than by their partner^48^, and even less by a stranger^12,19^. It is also well documented that humans are more prone to exchange social touch with people with whom they share a close and intimate relationship^48^ and that the beneficial effects of being touched are present if the giver is a romantic partner^16,18^. However, tactile affective exchange is a mutual dyadic behavior, and our results complement previous studies that have reported beneficial effects of *affective touch* only from the receiver’s point of view.

Additionally, we not only found that promoting *affective touch* to the partner was perceived as more pleasant than promoting it to a stranger but also that the former reduced anxiety level compared to the latter. Participants reported lower levels of anxiety after promoting *affective touch* to their partner compared to a stranger, thus confirming that the beneficial calming effects of *affective touch* between romantic partners are present also in the person who promotes the touch. Indeed, receiving *affective touch* from a partner helps regulating emotional responses^14^, increases the feeling of being supported during distress^16^, and attenuates pain perception^17^. Thus, these results suggest that also in the person promoting *affective touch* the anxiety relief might be closely linked to a boost in affectivity, well-being, and bonding^49^ between romantic couples.

The link between hedonic and calming effects of *affective touch* seems to rely on the role of specific afferent fibers, the C-Tactile (CT), which have been argued to mediate both pleasure^50^ and stress reduction effects during *affective touch*^51^*.* CT fibers take a direct ascending pathway from the periphery to the posterior insula^3,52^ and *affective touch* on CT-rich skin has been shown to elicit the activation of the posterior insula among other social-brain areas^53^. The posterior insula also plays a central role both in autonomic regulation^54^ as well as in the affective component of the touch^3^ by recruiting the central reward system^55^. Altogether, these elements provide strong evidence in support of the idea that CT fibers might play a central role in linking the social meanings of *affective touch* with its hedonic and anxiolytic effects.

Interestingly, while only hairy skin is rich in CT afferents, stimulation of glabrous skin (i.e., the stroking hand) can produce pleasant sensations as well. For example, stroking smooth, soft surfaces produces a pleasant effect^56^, and *affective touch* in glabrous skin has been found to activate several brain structures also related to the CT-mediated pleasant aspects of touch^53^. Moreover, studies on social softness illusion showed that people find it more pleasant to touch another's skin than their own^36^. Importantly, such effect only occurs when stimulation follows CT-optimal speed^36^ and is stronger when is provided in allocentric position^35^. Not surprisingly, people also show a natural tendency to stroke a beloved one at CT-optimal velocities^57^. Taken together, these observations emphasize the social nature of *affective touch* and support the idea that the giver’s hedonic experience might rely on a learned affective meaning associated to the positive experience of being stroked over CT-innervated skin. Consistently with the social nature of *affective touch*, in the present study we bring evidence that the pleasantness in promoting *affective touch* can be drastically modulated by two key contextual social factors: the identity of the person receiving the touch and the presence of an eye contact exchange. Indeed, touching the partner was perceived as more pleasant than touching a stranger, especially if the touch occurred together with direct eye contact. Our results are therefore in line with the concept that promoting *affective touch* can be a pleasant experience and extend this idea by showing that the degree of pleasantness also depends on specific dyadic contextual factors.

Finally, although most studies have avoided investigating the role of visual feedback between participants during *affective touch*, for example by separating participants with a curtain ^15,36^, we sought to understand how such variable might impact the experience of the person promoting *affective touch.* We found that when *affective touch* was accompanied by a mutual eye contact it further enhanced its pleasantness. Eye contact has been argued to play a major role in strengthening emotional sharing between individuals and evoking positive affective reactions^28^. In fact, the meaning of an eye contact is subordinated to contextual factors^29,58^ and a direct gaze exchange with a familiar person can evoke positive affective reactions^31^. Thus, the enhanced pleasantness reported when *affective touch* is combined with an eye contact could trigger an intrinsic hedonic reward and reinforce the motivational tendency to engage with a beloved one in an affective interaction.

### Physiological responses

Together with the subjective hedonic experience and self-report anxiety level, we investigated the physiological responses by recording and tracking skin conductance and heart rate in the person promoting *affective touch.* We found that caressing the partner compared to a stranger resulted in a general lower skin conductance activity. Skin conductance is a well-known indicator of autonomic arousal associated to affective states^38^; thus, our results could reflect a lower level of arousal when caressing the partner and an increased arousal with a stranger. Previous studies have reported several positive effects of *affective touch* between two partners both at the psychological^49^ and physiological level^18^. Although based on the person receiving *affective touch,* these observations have suggested that touch influences homeostatic modulation and conveys social meanings such as closeness and intimacy^59^, by promoting positive effects on the person receiving it by eliciting C-Tactile fibers^37^. However, other studies also suggested that, at an inter-individual level, touch alone can mediate a co-adaptation of autonomic activities between interacting individuals^18^ and thus may influence the giver as well. In line with this possibility, we found evidence that the modulating effect of *affective touch* on arousal is not restricted to the person receiving it, but it can also function as an input capable of modulating the physiological state of the giver itself. Additionally, to rule out that the physiological responses observed were not driven by any anticipatory effect, we conducted a second experiment and found both an independent and stronger autonomic response during *affective touch* as compared to the period immediately preceding it.

Interestingly, we observed a gender asymmetry in autonomic responses in the person promoting *affective touch*. Both skin conductance and heart rate activity suggested that for females, but not males, stroking the partner reduces the arousal whereas *affective touch* promoted to a stranger increases autonomic responses. Similarly to skin conductance, a lower heart rate corresponds to a lower state of distress^39^; hence, our results strongly suggest that females benefit from the calming effect of promoting *affective touch* to their partners. These physiological measures are supported by self-report anxiety states (STAI-Y1 questionnaire) which indicated that only females experienced an increase in anxiety after promoting *affective touch* to a stranger and a decrease in anxiety after the interaction with their partner. The gender effect observed in our study aligns with previous findings reporting that touch from an opposite-sex stranger is more avoided^60^ and perceived as more unpleasant by women than by men who, on the contrary, report it to be a quite pleasant experience^22^. Thus, our results are in line with what has been previously showed, providing new evidence on gender differences at the physiological level also when *affective touch* is promoted.

Differences at the physiological levels were also observed when we manipulated the presence of mutual eye contact between the two participants. Indeed, *affective touch* and face-to-face interaction are two central elements of social exchanges^32^ and eye contact itself has already been shown to have a strong communicative and affective value^28,61^ and to increase physiological activation^28,43,62,63^. We found that during an affective tactile interaction the physiological effects of an eye contact strongly depend on the relationship between the two people involved, with a greater physiological activation with a stranger than the partner. A direct eye contact with a stranger can evoke unpleasant and stressful reactions^29,31,58^ and therefore increase arousal levels. Thus, an eye contact interaction with a stranger might be processed as a potential social threat hence eliciting rapid sympathetic responses, whereas an eye contact with a partner might be processes as a calming source of well-being reflecting a general down-regulation of autonomic alertness by readily recruiting a parasympathetic activation. As in everyday life *affective touch* often occurs together with an eye contact exchange, with our second experiment we ruled out the possibility that autonomic responses when promoting *affective touch* to either the partner or a stranger could be driven uniquely by an eye contact exchange. Indeed, we found that *affective touch* produced a larger physiological response than eye contact alone.

There are several limitations we should acknowledge as well as possible future directions for new investigations. In our study, we only used a specific interpersonal touch which is *affective touch.* A comprehensive understanding of the effects, on the giver, of different kinds of social touch such as handholding, a handshake as well as deep pressure touch^64^ is still lacking. Future research should enlarge the present findings and investigate the effects at the subjective and autonomic level of different kinds of touch on the person promoting it. Furthermore, here we decided to enroll solely heterosexual couples to limit the number of variables at play; however, the hedonic and autonomic responses of the person promoting *affective touch* might change if interacting with a same-sex individual. Indeed, in our study we observed a gender asymmetry; in females, but not in males, stroking the partner reduced anxiety and arousal whereas *affective touch* promoted to a stranger increased autonomic response. Hence, the field would benefit from the investigation of the hedonic and autonomic responses of same-sex individuals. Finally, in our study, both *affective touch* and eye contact lasted 36 s. We adopted a fixed amount of time in order to directly compare different conditions; however different durations of either *affective touch* or eye contact might have different meanings and therefore modulate a person’s experience^65,66^. It would be important for future research to vary the amount of time subjects spend engaging in a social interaction to survey and understand possible timing effects. Finally, this study has investigated how three social variables (i.e., interpersonal relationship, gender and visual feedback) could modulate both the subjective experience and the autonomic responses of the person promoting *affective touch*. However, many other factors such as personality traits, touch exposure, and cultural context might also play an important role. Future research considering all these factors could shed light on the influence of other factors on the experience of receiving or promoting *affective touch*.

In conclusion, the scientific relevance of the present study lies on several grounds. First, using a naturalistic paradigm and setting we have shown that  *affective touch* elicits autonomic reactions in the person promoting it, and not only in the person receiving it as previously shown. These findings highlight that the act of caressing a beloved one promotes a calming effect as well as an enhanced hedonic experience, and that such interaction is even more pleasant if accompanied by mutual eye contact. The autonomic system seems to encode and map the subjective experience with a greater decline in heart rate and skin conductance when caressing a partner compared to a stranger, which however are also modulated by both gender and mutual eye contact. On the other hand, interacting with a stranger while promoting *affective touch* not only has been reported to be less pleasant and to increase anxiety levels but it is also accompanied by a general increase in the arousal level, although mediated by gender and eye contact between the two interacting participants. These results bring a novel contribution to the social neuroscience field by suggesting that different social variables, such as the relationship between the two individuals, the gender of the person promoting the touch, and mutual eye contact are encoded by the autonomic nervous system and modulate the physiological responses in the person promoting *affective touch*.

## Methods

### Experiment 1

#### Participants

Fifty participants were recruited and participated to the study accompanied by his/her partner. Our sample included 25 females (M = 24,2 years, SD = 2.52) and 25 males (M = 25,24 years, SD = 2.65). A priori power analysis conducted in G*Power^67^ indicated that, for a 2 × 2 × 2 mixed-factors ANOVA with 2 groups and 4 repeated measurements, the minimal required sample size for reaching the power of 0.80 with a medium effect size (η_p_^2^ = 0.06) and alpha level = 0.05 was determined to be 24. Four confederates (2 females and 2 males) played the role of a stranger. The only inclusion criteria were being a male or a female between 20 and 30 years of age and being involved in a heterosexual relationship for at least six months. All participants were recruited from a participants’ database or through flyers posted on the University website and gave their written informed consent to participate in the study. All experimental procedures were approved by the Bioethical Committee of the University of Turin and conducted in accordance with the ethical standards of the 2013 Declaration of Helsinki. At the end of the experiment, all participants were informed about the aims and scopes of the experiment and did not receive any compensation for participation in this research study.

#### Experimental design, setting and task

During the experimental session participants sat in front of each other diagonally shifted in allocentric position (Fig. 1a). The receiver’s right forearm was positioned on the table so that the giver could promote *affective touch* with his/her dominant hand. The giver was facing a computer screen located approximately 80 cm away, where trial-by-trial instructions were displayed. Before the beginning of the experiment two Ag–AgCl non-polarizable electrodes were attached to the medial phalanges of the index and the ring fingers of the non-dominant hand by velcro straps. Electrodermal activity (EDA) was recorded for the whole experimental session by using an MP160 biosignal amplifier working with a specific acquisition module for electrodermal activity EDA100-C (Biopac Systems, Inc.). The gain parameter was set at 10 µSiemens (µS)/Volt and the signal sampled at 500 Hz with a 0.05 Hz high pass filter. Additionally, electrocardiogram (ECG) pre-gelled shielded electrodes were applied with a Lead II montage using the standard limb electrode placement^68^. ECG was recorded by using an MP160 biosignal amplifier working with a specific acquisition module for electrocardiogram ECG100-C (Biopac Systems, Inc.). The gain parameter was set at 1000 (± 10 mV), and the signal sampled at 500 Hz with a 150 Hz low pass and a 0.05 Hz high pass filters.

The experimental session was divided into two blocks in which participants (i.e., the givers) promoted *affective touch* to a receiver. In one block the receiver was the giver’s partner while in the other block the receiver was a stranger (an opposite-gender confederate; Fig. 1b). Each block consisted of 18 consecutive trials. In 8 trials the participant was asked to deliver the *affective touch* to the receiver while looking at him/her in the eyes for the whole duration of the trial (Eye Contact condition); in another 8 trials, the participant was asked to deliver the *affective touch* to the receiver while looking at a fixation cross on the screen for the whole duration of the trial (Non-eye Contact condition; Fig. 1c). The remaining two trials were catch-trials in which participants were asked to touch their own forearm, with the aim of preventing habituation to hetero-directed touch.

At the beginning of each trial the giver received on the screen the instructions for the upcoming touch (e.g., “Touch with Eye Contact”) together with a 5 s count-down. Then, a 36 s fixation cross appeared on the screen and during this time the giver was invited to promote *affective touch*. Next, a Visual Analog Scale (VAS) was presented on the screen for 14 s, and the subject had to rate the pleasantness of the touch he/she had promoted on a scale ranging from −10 (Unpleasant) to +10 (Pleasant) by means of the computer mouse. Lastly, at the end of each trial a 5 s black screen was shown on the computer monitor, representing the inter-trial interval (ITI) (Fig. 1d).

#### Procedure

After being introduced to the study, each participant read and signed the consent form. Before the experimental session started, the area of touch (approximately 24 cm) was marked on both partners’ and confederates’ forearms. The touch was performed with the dominant hand (with index, middle and ring fingers) and executed in form of stroking movements with a speed of approximately 4 cm/s, which is the speed known to promote *affective touch*^37^. Participants were exposed to a training session to perform a light bidirectional touch over the forearm of a same-sex experimenter (from the end of the forearm to the wrist and in the opposite direction) with the help of a metronome, ringing every 6 s to indicate when to reverse the direction of touch. The training session consisted of four example trials and ensured that participants could familiarize with stroking at CT-optimal speed. Importantly, the metronome was also present along the whole duration of each experimental session, ensuring that participants delivered their strokes at approximately 4 cm/s in all trials. Following the training, each subject completed two experimental blocks: one with his/her partner and one with a stranger (confederate). At the end of each block participants were asked to fill in the STAI-Y1 questionnaire (see “Questionnaires” section for details) to assess changes in anxiety levels after promoting a touch to the partner (*Post Partner*) and after promoting a touch to a stranger (*Post Stranger*), compared to the beginning of the experimental session (Fig. 1e). The experiment had a total duration of about 40 min for each participant. Within each block, the order of trials presentation was pseudo-randomized, and the order of experimental blocks presentation (i.e., one with the partner and one with a stranger) was counterbalanced across participants.

#### Questionnaires

Before the experimental session started, participants were required to fill out four self-report questionnaires: the Social Touch Questionnaire (STQ), a 20 items questionnaire that explores the personal attitude toward social situations involving touch in everyday life^69^; the Social Interaction Anxiety Scale (SIAS), a 19 items questionnaire that measures distress when meeting and talking with others ^70^; the Dyadic Adjustment Scale (DAS), a 32 items questionnaire that measures an individual’s perceptions of their relationship with an intimate partner^71^; and the State-Trait Inventory (STAI-Y1) a 40 items questionnaire assessing anxiety state ^72^. The STAI-Y1 was filled two additional times during the whole experiment: after the block with the partner and after the block with the stranger (see below for details) to assess change in anxiety levels when participants promoted *affective touch* to a stranger compared to their partner. However, we also took advantage of other three self-report (STQ—SIAS—DAS) to make sure that any differences observed at the hedonic and physiological level among participants were not driven by a different attitude or a general different subjective profile.

#### Data processing and data analyses

##### Hedonic subjective measures

Pleasantness ratings from the giver (VAS) were analyzed with a 3-way mixed-factors ANOVA with Other (Partner vs Stranger) and Gaze (Eye contact vs Non-eye Contact) as within-subject factors and Gender (Males vs Females) as between-subject factor. Significant interactions were followed up by Bonferroni-corrected t-tests for either dependent (Other and Gaze factors) or independent (Gender factor) samples and values of p < 0.05 were considered significant.

##### Autonomic measures—skin conductance

The EDA data were filtered online using a 0.05 Hz high pass filter, then processed and analyzed offline using Matlab (release 2021a, The MathWorks, Inc.). Raw data were linearly detrended and numerically zeroed by subtracting the minimum EDA value within each recording window^73^. Next, we extracted the mean of the signal as an indicator of phasic skin conductance activity occurring during the epoch^74,75^. Lastly, the extracted mean values were normalized within each subject by means of an ipsatization^76^ to control for interindividual variability: for each trial, the analyzed physiological measure was z-scored by subtracting the mean of all trials in all conditions for a given subject and divided by the standard deviation of all trials and conditions for that subject. Within-subject z-transformations have the advantage of minimizing the impact of outlier values, because means are centered at zero and the within-subject variability is put on a common metric^76,77^. We analyzed the EDA activity for each trial (0–36 s) with a 3-way mixed-factors ANOVA with Other (Partner vs Stranger) and Gaze (Eye contact vs Non-eye Contact) as within-subject factors and Gender (Males vs Females) as between-subject factor. Significant interactions were followed up by Bonferroni-corrected t-tests for either dependent (Other and Gaze factors) or independent (Gender factor) samples and values of p < 0.05 were considered significant.

##### Autonomic measures—heart rate

ECG heart rate (Beats Per Minute, BPM) data were averaged separately for each block (i.e., partner and stranger) and the recording time was 18 min for each block. For this interval and for each participant, the mean heart rate was calculated. For BPM, 4 out of 50 participants (i.e., 2 couples) were excluded from analyses due to bad sensor data. Data were normalized within each subject by means of an ipsatization^76^, as reported above for EDA measures. BPM changes were analyzed with a 2-way mixed-factors ANOVA with Other (Partner vs Stranger) as within-subject factor and Gender (Males vs Females) as between-subject factor. Significant interactions were followed up by Bonferroni-corrected t-tests for either dependent (Other factor) or independent (Gender factor) samples of p < 0.05 were considered significant.

##### Subjective measures

In order to assess the overall changes in state anxiety levels after promoting *affective touch* to the partner or to a stranger, we computed the difference (delta, Δ) between the respective post-block STAY-Y1 and the baseline STAY-Y1. This delta was computed as *STAI-Y1*_*(Post Stranger)*_*−STAI-Y1*_*(Baseline)*_ for the stranger and as *STAI-Y1*_*(Post Partner)*_*−STAI-Y1*_*(Baseline)*_ for the partner. Thus, positive values indicate an increase and negative ones indicate a decrease in anxiety state compared to the baseline. To investigate any difference in state anxiety changes, we ran a 2-way mixed-factors ANOVA with Other (Δ_Partner_ vs Δ_Stranger_) as within-subject factor and Gender (Males vs Females) as between-subject factor. Also, to explore any increase or decrease in the anxiety level after each experimental block, we compared each of the four conditions against zero. One female subject was excluded from analyses due to missing data.

### Experiment 2

#### Participants

Twenty participants were recruited for this experiment accompanied by his/her partner. Data of two participants were missing due to technical failures in data recording, and our final sample size was N = 18, including 10 females (M = 23,10 years, SD = 3.66) and 8 males (M = 23,12 years, SD = 3.52). A priori power analysis conducted in G*Power^67^ indicated that, for a 2 × 2 × 2 mixed-factors ANOVA with 2 groups and 4 repeated measurements, the minimal required sample size for reaching the power of 0.80 with an effect size η_p_^2^ = 0.135 and alpha level = 0.05 was determined to be 12. As in Experiment 1, four confederates (2 females and 2 males) played the role of a stranger. The only inclusion criteria were being a male or a female between 20 and 30 years of age and being involved in a heterosexual relationship for at least six months. All participants were recruited from a participants’ database or through flyers posted on the University website and gave their written informed consent to participate in the study. All experimental procedures were approved by the Bioethical Committee of the University of Turin and conducted in accordance with the ethical standards of the 2013 Declaration of Helsinki. At the end of the experiment, all participants were informed about the aims and scopes of the experiment and did not receive any compensation for participation in this research study.

#### Experimental design, setting, and task

The experimental setting was similar to the one described in Experiment 1. Before the beginning of the experimental session, we positioned electrodes for recording EDA from the giver in the same locations and with the same parameters used in Experiment 1. The experimental session was divided in two blocks. In one block, the receiver was the subject’s partner, whereas in the other block the receiver was a stranger (an opposite-gender confederate) (Fig. 1b). Each block consisted of 20 consecutive trials. Because we were interested in investigating the differences at both the hedonic and physiological level between eye contact and *affective touch*, in 9 trials participants were asked to interact with the receiver by promoting *affective touch* while looking at him/her in the eyes for the whole duration of the trial (Touch + Eyes condition) (Fig. 4a), while in other 9 trials participants were asked to interact with the receiver by merely engaging with eye contact for the whole duration of the trial (Eyes-Only condition) (Fig. 4b). The remaining two trials were catch-trials in which the participants were asked to look at a fixation cross on the screen (on one trial) or to stroke their forearm while looking at a fixation cross on the screen (on the second one).

At the beginning of each trial the giver received on the screen the instructions for the upcoming trial. Instructions could either last 5, 10 or 15 s, and no visual countdown was presented on the screen (Fig. 4c). Thus, in each trial participants could not predict when the experimental condition would have started. Participants were instructed to start the trial as soon as instructions disappeared from the screen. Then, similarly to Experiment 1, a 36 s fixation cross appeared on the screen: during this time, the giver was invited to either promote *affective touch* while looking at the receiver in the eyes (Touch + Eyes condition) or to just engage in a mutual eye contact with the receiver, without promoting any *affective touch* (Eye Contact-only condition). Next, a Visual Analog Scale (VAS) was presented on the screen for 14 s, and the subject was asked to rate the pleasantness of the *affective touch* or eye contact he/she had promoted on a scale ranging from −10 (Unpleasant) to +10 (Pleasant) by means of the computer mouse. Lastly, a 5 s black screen representing the inter-trial interval (ITI) was shown at the end of each trial (Fig. 4b).

#### Procedure

After being introduced to the study, each participant read and signed the consent form. Then we asked participants to fill in the STAI-Y1 questionnaire for assessing their anxiety state. The STAI-Y1 was filled two additional times during the whole experiment: after the block with the partner and after the block with the stranger (see below for details). Before the experimental session started, the area of touch (approximately 24 cm) was marked on both partners’ and confederates’ forearms. The touch was performed with the dominant hand (with index, middle, and ring fingers) and executed in form of stroking movements with a speed of approximately 4 cm/s. Participants were familiarized to perform a light touch, lasting 36 s, over the forearm of a same-gender experimenter bidirectionally with the help of a metronome, ringing every 6 s and indicating to reverse directionality of touch (from the end of the forearm to the wrist and in the opposite direction). Crucially the metronome was also present along the whole duration of each experimental session, ensuring that participants delivered their strokes at approximately 4 cm/s (CT-optimal speed range^37^]) in all trials. They were also familiarized to perform an eye contact with the experimenter that lasted for 36 s with the help of a metronome (the same used for *affective touch* condition). At the end of each block participants were asked to fill in the STAI-Y1 to assess change in anxiety levels, compared to the beginning of the experimental session, after interacting with the partner (*Post Partner*) and with a stranger (*Post Stranger*).

The experiment had a total duration of about 40 min for each participant. Within each block, the order of trials presentation was pseudo-randomized, and the order of experimental blocks presentation (i.e., one with the partner and one with a stranger) was counterbalanced across participants.

#### Data processing and data analyses

##### Hedonic subjective measures

Pleasantness ratings from the giver (VAS) were analyzed with a 3-way mixed-factors ANOVA with Other (Partner vs Stranger) and Touch (Touch + Eyes vs Eyes Only) as within-subject factors and Gender (Males vs Females) as between-subject factor. Significant interactions were followed up by Bonferroni-corrected pairwise t-tests for independent samples and values of p < 0.05 were considered significant.

##### Autonomic measures—skin conductance

As in Experiment 1, we extracted the mean EDA value of the signal from 0 to 36 s and ipsatized those. Data were then analyzed with a 3-way mixed-factors ANOVA with Other (Partner vs Stranger) and Touch (Touch + Eyes vs Eyes Only) as within-subject factors and Gender (Males vs Females) as between-subject factor. Significant interactions were followed up by Bonferroni-corrected t-tests for either dependent (Other and Touch factors) or independent (Gender factor) samples and values of p < 0.05 were considered significant.

To investigate the presence of any anticipatory effects in trials where *affective touch* was promoted (Touch + Eyes condition), we measured the onset latency of the first peak during *affective touch*. For each trial we extracted the latency of the first peak occurring during the experimental condition (i.e., after the instructions had disappeared from the screen), and then measured when the first preceding trough occurred, i.e., where the same peak had started. Thus, if the peak had started raising *before* the beginning of the *affective touch*, the peak itself would have represented an anticipation-driven phasic response leaking into the trial period, making it a spurious response. On the contrary, if the peak had started raising *after* the beginning of the *affective touch*, it would have represented a pure trial-driven phasic EDA response. Our hypothesis was that the peak would have begun rising only after the beginning of the trial, independently of the duration of the instructions. To test this hypothesis, we ran three separate one-tail 1-sample t-tests, one for each level of instructions duration as we aimed at excluding any anticipatory effect of touch and eye contact in Experiment 1. Peak onset times in 5, 10, and 15 s instructions condition were contrasted against zero (the end of the instructions period). Peak detection threshold was set to 0.01 µS.

Also, to investigate whether EDA activity during *affective touch* was not only independent from, but also larger than the activity during the task instructions, we extracted the maximum value of the EDA signal during the instructions and during *affective touch* period (Touch + Eyes condition). For trials with 5 s baseline, we extracted the whole 5 s baseline signal and the first 5 s of the signal during *affective touch*; the same approach was adopted for trials with 10 s and 15 s instructions. Then, the difference *Max*_*AffectiveTouch*_*−Max*_*Instructions*_ was calculated in each time conditions (i.e., 5, 10 and 15 s instructions duration). Deltas were then contrasted against zero for both trials with Partner and Stranger, independently from baseline duration—i.e., aggregating trials with 5, 10, and 15 s instructions duration. Our hypothesis was to observe positive deltas significantly different from zero, indicating a larger EDA signal during *affective touch* than during instructions.

##### Subjective measures

In order to assess the overall changes in state anxiety levels after promoting *affective touch* to the partner or to a stranger, as we have done for Experiment 1, we computed the difference between the respective post-block STAY-Y1 and the baseline STAY-Y1. Thus, positive scores indicate an increase in state anxiety, negative scores indicate a decrease. We followed the same approach and analysis adopted in Experiment 1. Briefly, we first ran 2-way mixed-factors ANOVA with Other (Partner vs Stranger) as within-subject factor and Gender (Males vs Females) as between-subject factor. Next, we compared conditions against zero to investigate a significant increase or decrease compared to baseline.

## Supplementary Information

Supplementary Information.

## Data Availability

Behavioral and physiological data presented in this paper will be available upon request to the corresponding author (olga.dalmonte@unito.it).
