# Supplementary material for: Hedonic and autonomic responses in promoting affective touch
Source: Sci Rep. 2023 Jul 11;13:11201. doi: 10.1038/s41598-023-37471-9 (PMC10336056; doi:10.1038/s41598-023-37471-9)
Supplement: Supplementary file 1 — Supplementary Information. [file 41598_2023_37471_MOESM1_ESM.docx]

**Supplementary Information**

**What touching you makes me feel - hedonic and autonomic responses in promoting affective touch**

Authors:

Alessandro Mazza^1^, Monia Cariola^1^, Francesca Capiotto^1^, Matteo Diano^1^, Selene Schintu^2,3^, Lorenzo Pia^1^, and Olga Dal Monte^1,4^

Affiliations:

1 Department of Psychology, University of Turin, Torino, Italy

2 Center for Mind/Brain Sciences-CIMeC, University of Trento, Rovereto, Italy.

3 Department of Psychology, The George Washington University, Washington DC, DC, USA

4 Department of Psychology, Yale University, New Haven, CT, USA

Supplementary information:

Supplementary Figure S1-S3

Supplementary Results

**Supplementary Figure 1**

**
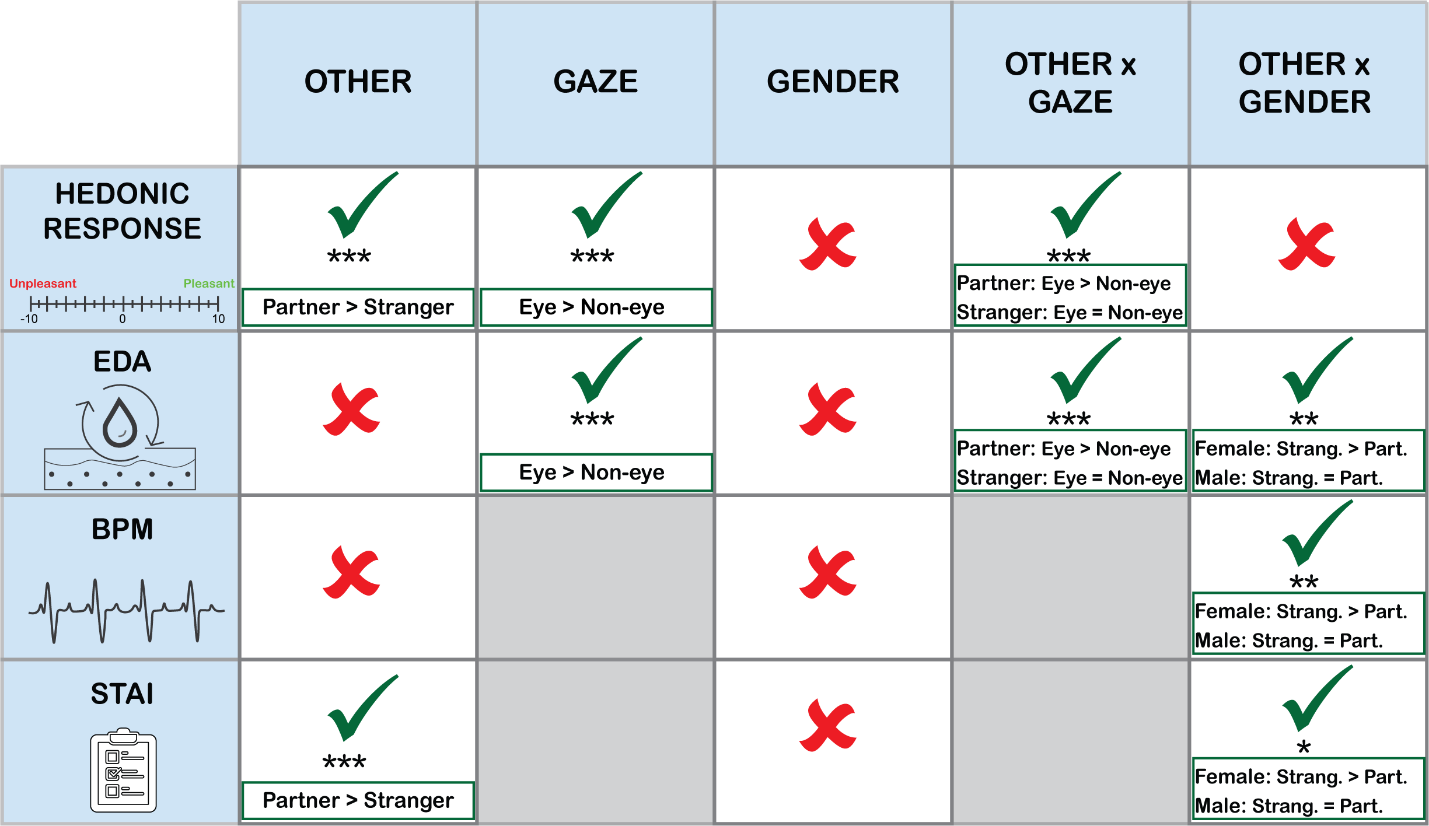
**

**Supplementary Figure 1. Results summary Experiment 1.**

Rows depict Hedonic Response, Electrodermal Activity (EDA), Heart Rate (Beats Per Minute, BPM) and State-Trait Anxiety Inventory (STAI). Columns depict experimental variables: Other (Partner vs Stranger), Gaze (Eye contact vs Non-eye Contact) and Gender (Males vs Females) as well as their interactions (Other x Gaze and Other x Gender). Significant results are indicated by green check marks with * = p < 0.05; ** = p < 0.01; *** = p < 0.001. Not significant results are indicated by red crosses. Grey cells represent factors not included in a given analysis.

**Supplementary Figure 2**

**
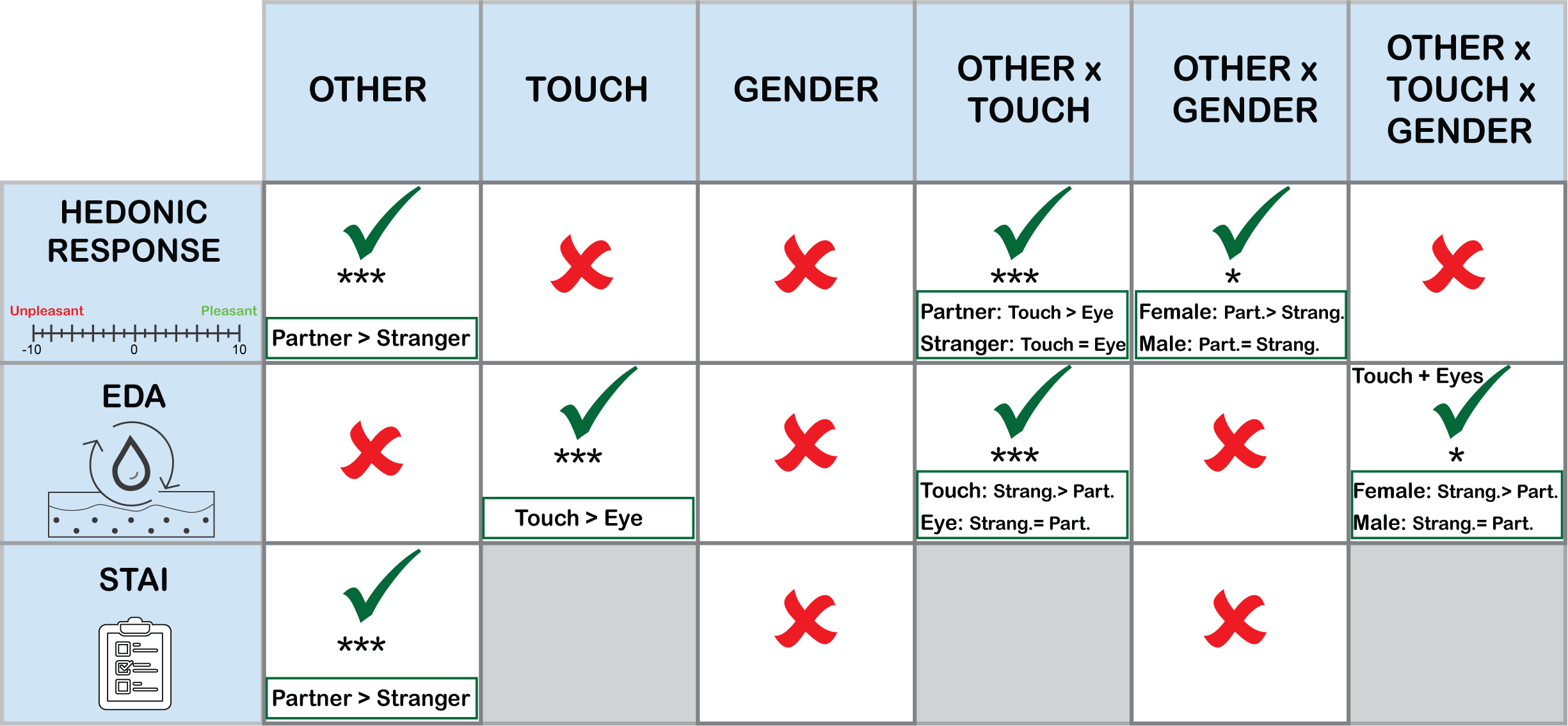
**

**Supplementary Figure 2. Results summary Experiment 2.**

Rows depict Hedonic Response, Electrodermal Activity (EDA), and State-Trait Anxiety Inventory (STAI). Columns depict experimental variables: Other (Partner vs Stranger), Touch (Touch+Eyes vs. Eyes Only) and Gender (Males vs Females) as well as their interactions (Other x Touch, Other x Gender, and Other x Touch x Gender). Significant results are indicated by green check marks with * = p < 0.05; ** = p < 0.01; *** = p < 0.001. Not significant results are indicated by red crosses. Gray cells represent factors not included in a given analysis. For EDA, the triple interaction (Other x Touch x Gender) shows that gender differences were observed only for the experimental variable Touch+Eyes: a larger EDA activity with a stranger than with the partner was observed in Females but not in Males. This effect was not present in Eye-Only condition.

**Experiment 2 - Supplementary Results**

To confirm that the increase in skin conductance observed during affective touch was related to the touch and not driven by any anticipatory effects due to the instructions given to the participants, in Experiment 2 we randomly varied the instructions time (5, 10, and 15 seconds) and removed any count-down from the monitor, so that participants could not predict the beginning of the touch. We targeted the first peak occurring after the end of the instructions period (i.e., during affective touch) and then tested whether the immediately preceding trough (i.e., the peak onset) occurred before or after the beginning of the affective touch (time zero), independently of the duration of the instructions. For all the three different timing used (5, 10, and 15 seconds) the peak onset occurred significantly after the beginning of the affective touch [for 5 seconds instructions: t(17) = 4.48, p < 0.001 [mean = 1.25 sec; 95% confidence interval (CI) = ± 0.59 sec]; for 10 seconds instructions: t(17) = 3.89, p < 0.001 (1.33 sec ± 0.72 sec); for 15 seconds instructions: t(17) = 3.40, p = 0.002 (1.71 sec ± 1.06 sec)]. Similar results were observed when the analyses were performed separately for Partner and Stranger (example trials are displayed in **Supplementary** **Figure 3a-b**). These results show that phasic EDA activity during the trial is independent from EDA activity during the instructions and demonstrate that in our paradigms the skin conductance response during affective touch is not driven by any anticipatory effect.

Also, we asked whether the skin conductance responses observed during the affective touch were not only independent from, but also larger than, those observed during the instruction’s presentation. For each instructions time (5, 10, and 15 seconds) we calculated the difference between mean EDA activity during the affective touch and during the instructions period. The t-test on the difference between affective touch-related activity and instructions-related activity with the Partner showed that this difference was significantly larger than zero [t(17) = 3.12, p = 0.003] (**Supplementary** **Figure 3c**); the same was true for the trials with affective touch delivered to the Stranger [t(17) = 3.34, p = 0.002] (**Supplementary Figure 3d**). These results show that the physiological responses are greater during the promotion of an affective touch than during the baseline. Thus, our findings suggested both an independent and a stronger autonomic response during an affective touch as compared to the instructions period.

**Supplementary Figure 3**


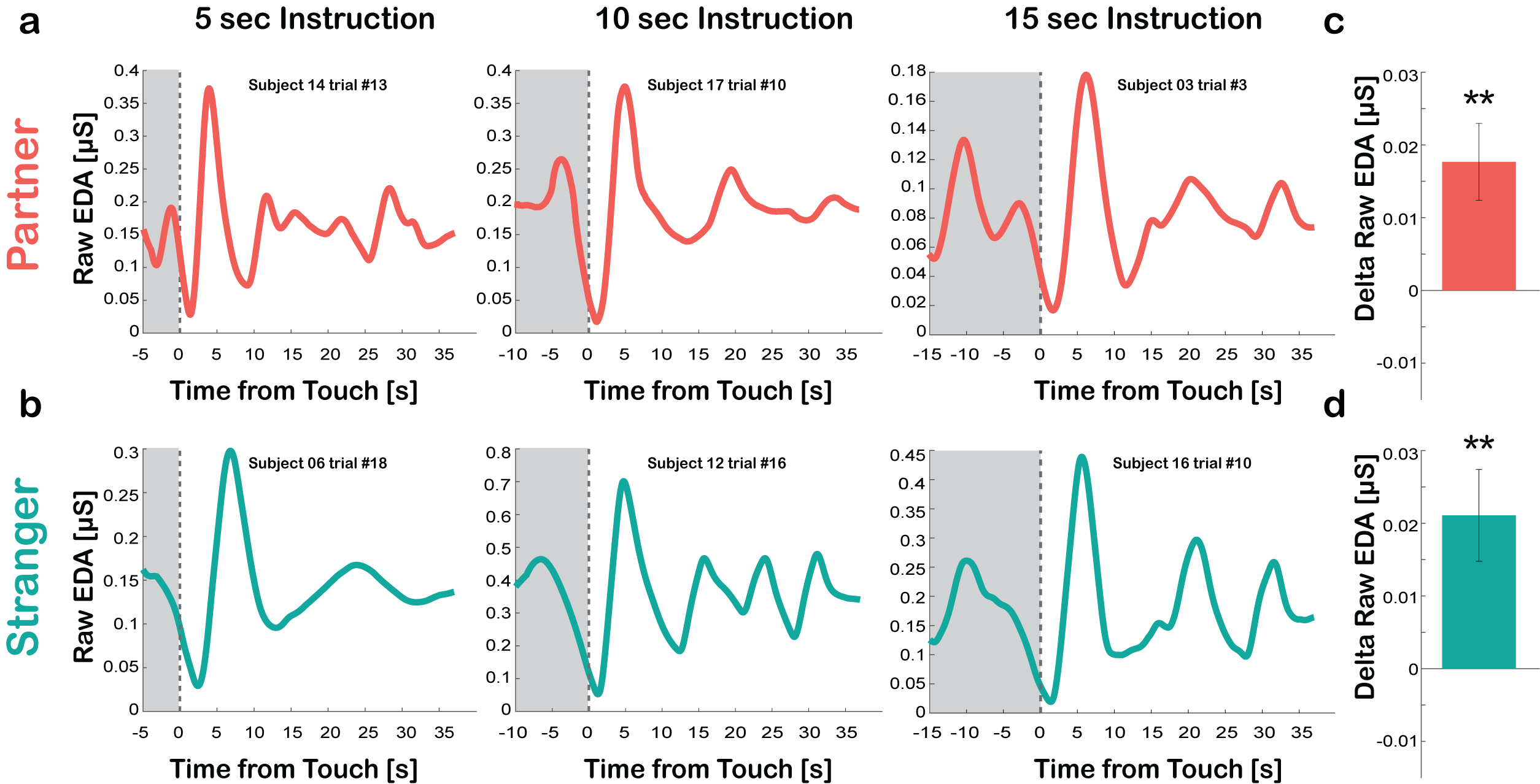


**Supplementary Figure 3. Electrodermal activity during the instructions and during affective touch.**

(a) Single-trace examples of raw EDA activity aligned to the time of affective touch with the Partner. Vertical dotted grey line indicates the beginning of affective touch period (from 0 to 36 second). Shaded area represents period after instructions onset (5, 10, and 15 second). (b) Single-trace examples of raw EDA activity aligned to the time of affective touch with a Stranger, same format as b. (c) Delta of raw mean EDA values during affective touch with the Partner minus the raw EDA activity during the instructions (d) Delta of raw mean EDA values during affective touch with a Stranger minus the raw EDA activity during the instructions. Values shown are the mean ± s.e.m. Significant results are indicated by asterisk ** = p < 0.01.
